# Machine learning-based prediction of 5-year survival in elderly NSCLC patients using oxidative stress markers

**DOI:** 10.3389/fonc.2024.1482374

**Published:** 2024-10-24

**Authors:** Hao Chen, Jiangjiang Xu, Qiang Zhang, Pengfei Chen, Qiuxia Liu, Lianyi Guo, Bindong Xu

**Affiliations:** ^1^ Department of Thoracic and Cardiovascular Surgery of the Affiliated Hospital of Putian University, Putian, Fujian, China; ^2^ Fuding Hospital, Fujian University of Traditional Chinese Medicine, Fuding, Fujian, China; ^3^ Department of Gastroenterology, The First Affiliated Hospital of Jinzhou Medical University, Jinzhou, China

**Keywords:** elderly, NSCLC, oxidative stress, machine learning, overall survival

## Abstract

**Background:**

Oxidative stress plays a significant role in aging and cancer, yet there is currently a lack of research utilizing machine learning models to examine the relationship between oxidative stress and prognosis in elderly non-small cell lung cancer (NSCLC) patients.

**Methods:**

This study included elderly NSCLC patients who underwent radical lung cancer resection from January 2012 to April 2018, exploring the relationship between Oxidative Stress Score (OSS) and prognosis. Machine learning techniques, including Decision Trees (DT), Random Forest (RF), and Support Vector Machine (SVM), were employed to develop predictive models for 5-year overall survival (OS).

**Results:**

The datasets consisted of 1647 patients in the training set, 705 in the internal validation set, and 516 in the external validation set. An OSS was formulated from six systemic oxidative stress biomarkers, such as albumin, total bilirubin, and blood urea nitrogen, among others. Boruta variable importance analysis identified low OSS as a key indicator of poor prognosis. The OSS was subsequently integrated into the DT, RF, and SVM models for training. These models, optimized through hyperparameter tuning on the training set, were then evaluated on the internal and external validation sets. The RF model demonstrated the highest predictive performance, with an Area Under the Receiver Operating Characteristic Curve (AUC) of 0.794 in the internal validation set, compared to AUCs of 0.711 and 0.760 for the DT and SVM models, respectively. Similarly, in the external validation set, the RF model achieved an AUC of 0.784, outperforming the DT and SVM models, which had AUCs of 0.699 and 0.730, respectively. Calibration plots confirmed the RF model’s superior calibration, followed by the SVM model, with the DT model performing the poorest.

**Conclusion:**

The OSS-based clinical prediction model, constructed using machine learning methodologies, effectively predicts the prognosis of elderly NSCLC patients post-radical surgery.

## Introduction

Non-small cell lung cancer (NSCLC) is a prevalent malignancy with a high incidence and mortality rate globally, particularly affecting the elderly population (1). Elderly cancer patients exhibit significant heterogeneity in physical, functional, psychological, and social dimensions, limiting the TNM staging system’s ability to accurately reflect their prognostic characteristics (2). Research has identified specific oxidative stress markers, including albumin (ALB), bilirubin, and uric acid (UA), that can induce malignant transformations in normal epithelial cells through various biological activities (3–5). Increased levels of reactive oxygen species and oxidative stress products have been observed in malignant tumor cells, playing a crucial role in tumor development and prognosis. On the other hand, the imbalance between the production and neutralization of oxidants in the elderly, along with reduced antioxidant enzyme activity, leads to pathological processes such as mitochondrial dysfunction, causing systemic disorders and thus predisposing to malignancies, cardiovascular diseases, neurodegenerative diseases, and more (6–9). In order to optimize treatment and improve quality of life for elderly NSCLC patients, clinicians should explore oxidative stress in these patients. Oxidative stress markers are not currently used to predict survival in elderly NSCLC patients.

The use of supervised machine learning methods in prognosis prediction is widespread due to their greater flexibility, especially when dealing with large and complex data sets (10–12). The majority of existing models rely on known variables like TNM staging and histological characteristics, which do not fully take into account the complex modifications that occur in the elderly. Considering the importance of oxidative stress in elderly NSCLC patients, this study aims to investigate the relationship between oxidative stress and prognosis. Moreover, it aims to create a machine learning model that can predict 5-year survival after surgery, thereby aiding in clinical decision-making.

## Methods

### Study population

**Figure 1:**
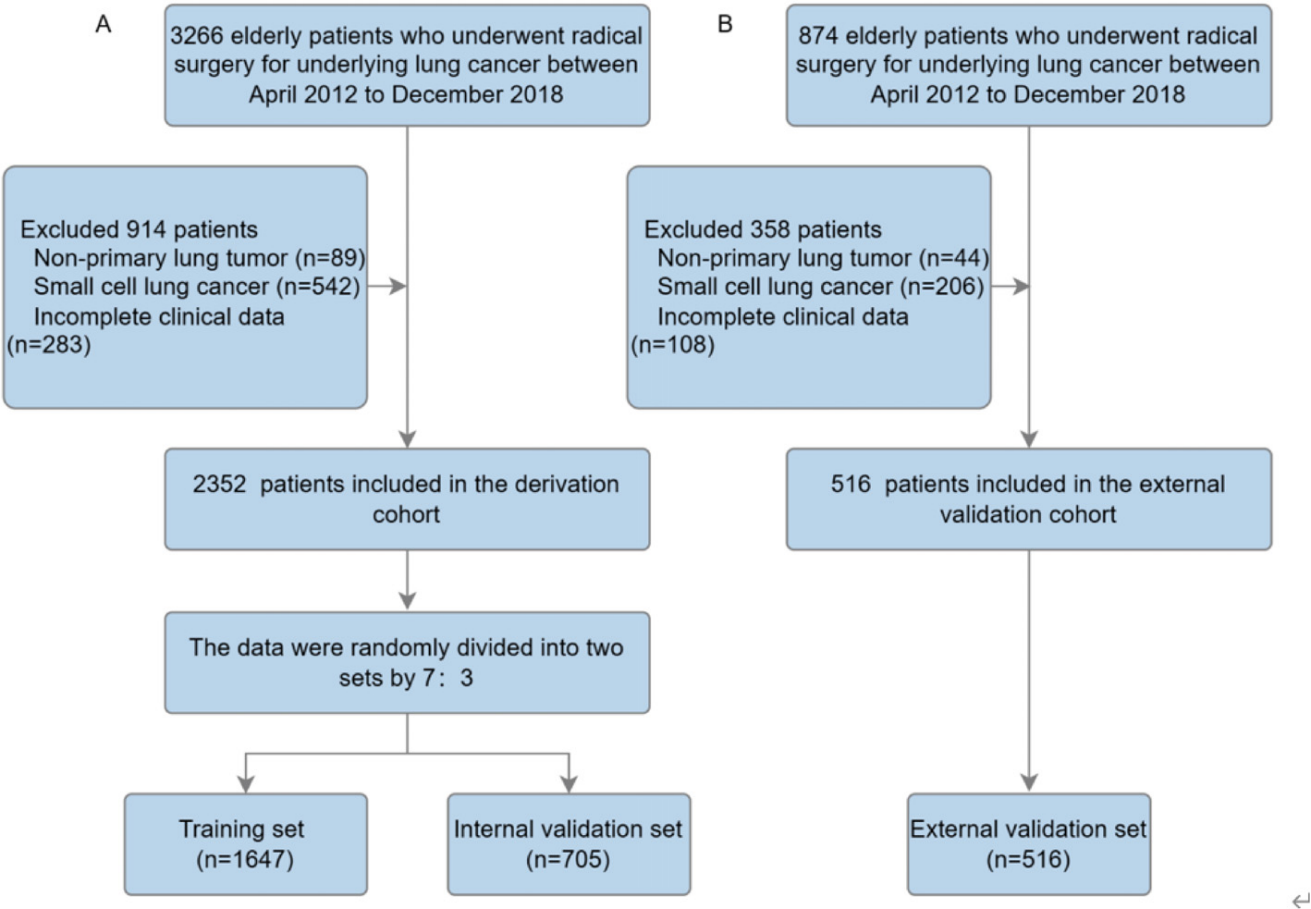
Study flow diagram. **(A)** Derivation set. **(B)** External validation set.

Based on the thoracic surgery database at the Affiliated Hospital of Putian University (AHPTH), 3,266 elderly lung cancer patients underwent radical lung cancer resection by Video-Assisted Thoracoscopic Surgery (VATS) between April 2012 and December 2018. This study included patients with (1) a postoperative pathological diagnosis of non-small cell lung cancer; (2) a diagnosis of age 65 or older; (3) radical surgical resection with no evidence of distant metastases; and (4) complete clinical and pathological data available. Exclusion criteria included: (1) tumors not originating in the lung; (2) postoperative pathology confirmed as small cell lung cancer; (3) incomplete clinical data. During the period of January 2012 to April 2018, 874 patients meeting the same inclusion criteria were included in the external validation cohort at Fujian University of Traditional Chinese Medicine. Ultimately, after exclusions, 2,352 patients were included in the derivation cohort, and 516 patients were included in the external validation cohort. The derivation cohort was randomly divided into two datasets in a 7:3 ratio: a training cohort (70%), used to train the three machine learning models and adjust their parameters, and an internal validation cohort (30%), used to test the developed models on unseen data and fine-tune hyperparameters (**Figure 1**) (13). Calibration curves and Area Under Receiver Operating Characteristic Curves (AUC) were used in the training, internal validation, and external validation cohorts to assess predictive performance. It was calculated that the time to event or censoring would be calculated from the date of surgery until the date of last contact (death or last follow-up). The institutional review board waived informed consent requirements because the research involved retrospective analysis of anonymized database data.

### Candidate predictive variables

Routine blood and biochemical tests were conducted from the day of admission for each patient, including preoperative tests, intraoperative conditions, postoperative recovery, and pathological results. In accordance with the 8th edition of the American Joint Committee on Cancer/Union for International Cancer Control (AJCC/UICC) Cancer Staging Manual, TNM staging has been reclassified. X-tile software was used to identify the best threshold values for categorizing biochemical markers. The oxidative stress markers studied included albumin (ALB), total bilirubin (TBIL), direct bilirubin (DBIL), urea (BUN), uric acid (UA), creatinine (Crs), and lactate dehydrogenase (LDH) were all conducted before surgery. According to the optimal threshold values, biochemical markers were classified as low or high (below or above the threshold). The training set was used to develop a new Oxidative Stress Score (OSS) based on variable coefficients in the Cox stepwise regression model combined with the Akaike Information Criterion (AIC). The best cut-off value of the OSS was used to stratify patients into different risk levels, and both internal and external validation cohorts validated this stratification (14, 15).

Other clinically relevant features for the machine learning predictive model were selected through a consensus among researchers, incorporating clinical reasoning, literature review, and routine availability. This approach ensures the model’s broad applicability across diverse clinical settings. Specifically, the variables selected for the predictive model encompassed a comprehensive range of clinical, preoperative, intraoperative, postoperative, and pathological factors. Clinical variables included gender, age, OSS, body mass index, Charlson comorbidity index, American Society of Anesthesiologists (ASA) score, smoking history, alcohol consumption history, history of diabetes, and history of pulmonary disease. Preoperative variables comprised hemoglobin, white blood cells, neutrophils, lymphocytes, fibrinogen, CEA, and CA125. Intraoperative variables included tumor location, tumor size, surgical time, and intraoperative blood loss. Postoperative variables covered Clavien-Dindo complication grading and adjuvant chemotherapy. Pathological variables involved the degree of differentiation, pathological type, pathological T stage, pathological N stage, and pathological TNM stage. To address potential collinearity, some predictors were excluded, opting for pathological T and N stages instead of the composite TNM stage. Variable standardization was conducted to ensure scale comparability.

### Construction and establishment of machine learning models

To predict the survival status at 5 years post-surgery, we analyzed the discriminative capabilities of three classification machine learning algorithms: random forest (RF), decision tree (DT), and support vector machine (SVM). These methods were chosen due to their widespread application and superior performance in cohort studies. All statistical analyses were conducted using several established R packages: “randomForest,” “MASS,” “PRROC,” “rpart,” “caret,” and “e1071.” To select the optimal hyperparameters and probabilities, models were trained using a cross-validation scheme. DT are supervised machine learning techniques used for both regression and classification tasks. DT predicts the target variable’s value by learning simple rules represented by a tree structure consisting of nodes, branches, and leaves. The algorithm classifies each sample by traversing the tree from the root to a leaf node. RF is an ensemble learning algorithm suitable for classification, regression, and unsupervised learning tasks. It consists of multiple unpruned decision trees created through a recursive partitioning process. Each tree in the forest is generated using the DT algorithm, enhancing the model’s overall accuracy and robustness. SVM is another widely used supervised learning algorithm for classification and regression tasks. SVM constructs one or more hyperplanes in a high-dimensional space to optimally separate data into different classes. For nonlinear classification problems, the Radial Basis Function (RBF) kernel is employed to estimate and maximize the margin between classes, enhancing the model’s performance in complex scenarios.

### Follow-up

Postoperative follow-up is recommended every 3-6 months within the first two years after surgery. From three to five years post-surgery, if the condition remains stable, follow-up visits are advised every 6-12 months. Beyond five years, annual check-ups are recommended. The follow-up includes tests such as complete blood count, biochemical markers, tumor markers, chest CT (with or without contrast), and ultrasound. Additional examinations may be conducted as needed based on the patient’s condition. The primary outcome was defined as overall survival (OS) after discharge, which was measured from the date of surgery to the date of death from any cause or to the last follow-up date for censored observations.

### Statistical analysis

Data analysis was conducted using R version 4.3.1. Differences in categorical variable distributions between groups were assessed with Pearson’s chi-squared test and Fisher’s exact test. Overall survival (OS) curves were generated using the Kaplan-Meier method, with the log-rank test used to evaluate differences between survival curves. Internal validation was carried out using bootstrap resampling. Model parameters were trained on the training dataset, and the performance of the trained models was evaluated using an independent validation dataset. The effectiveness of the trained classifiers was measured using Area Under the Curve (AUC) values, decision curves, and calibration curves.

## Results

### Study cohort

A total of 2,352 elderly lung cancer patients were included in the derivation cohort of this study, with 516 patients in the external validation cohort. The derivation cohort consisted of 1,368 males (58.16%) and 984 females (41.84%), with an average age of 71.33 ± 5.17 years. This cohort was randomly divided into a training set and an internal validation set in a 7:3 ratio. The external validation cohort comprised 307 males (59.5%) and 209 females (40.5%), with an average age of 71.6 ± 5.16 years. No significant differences in clinical or pathological data were observed between the training set and the internal validation set (**Table 1**, P > 0.05). While the external validation cohort showed a difference in the tumor marker CEA (P = 0.04), no significant differences were found in other variables compared to the training cohort (P > 0.05).

**Table 1:** Demographic and clinical characteristics of the derived cohort, training set, and internal validation set of elderly patients undergoing radical lung cancer surgery.

	Derivation set			External validation set	*P* value^b^
	Training set	Internal validation set	*P* value^a^		
n	1647	705		516	
Age (mean (SD))	71.35 (5.15)	71.28 (5.21)	0.767	71.60 (5.16)	0.294
Gender (%)					0.612
female	690 (41.9)	294 (41.7)	0.967	209 (40.5)	
male	957 (58.1)	411 (58.3)		307 (59.5)	
BMI (mean (SD))	22.38 (3.27)	22.61 (3.63)	0.12	22.40 (3.24)	0.795
ASA (%)			0.314		0.071
1	65 (3.9)	22 (3.1)		18 (3.5)	
2	1179 (71.6)	498 (70.6)		364 (70.5)	
3	401 (24.3)	182 (25.8)		129 (25.0)	
4	2 (0.1)	3 (0.4)		5 (1.0)	
Smoking history (%)			0.925		0.083
No	1196 (72.6)	514 (72.9)		355 (68.8)	
Yes	451 (27.4)	191 (27.1)		161 (31.2)	
Pulmonary Disease (%)			0.64		0.11
No	1603 (97.3)	683 (96.9)		494 (95.7)	
Yes	44 (2.7)	22 (3.1)		22 (4.3)	
CEA (mean (SD))	13.60 (72.06)	11.45 (56.28)	0.481	6.75 (21.05)	0.04
CA125 (mean (SD))	15.67 (71.04)	14.34 (31.62)	0.633	13.48 (14.92)	0.513
Diameter (mean (SD))	4.25 (2.52)	4.24 (2.40)	0.909	4.12 (2.38)	0.284
pT (%)			0.311		0.061
1	651 (39.5)	281 (39.9)		223 (43.2)	
2	442 (26.8)	189 (26.8)		145 (28.1)	
3	492 (29.9)	219 (31.1)		140 (27.1)	
4	62 (3.8)	16 (2.3)		8 (1.6)	
pN (%)			0.554		0.341
0	1041 (63.2)	460 (65.2)		339 (65.7)	
1	282 (17.1)	107 (15.2)		69 (13.4)	
2	201 (12.2)	91 (12.9)		70 (13.6)	
3	123 (7.5)	47 (6.7)		38 (7.4)	
pTNM (%)			0.696		0.34
I	605 (36.7)	870 (37.0)		208 (40.3)	
II	565 (34.3)	248 (35.2)		173 (33.5)	
III	477 (29.0)	192 (27.2)		135 (26.2)	
Tumor Histology (%)					0.061
Adenocarcinoma	1197 (72.7)	515 (73.0)	0.885	387 (75.0)	
Adenosquamous carcinoma	83 (5.0)	38 (5.4)		14 (2.7)	
Squamous carcinoma	367 (22.3)	152 (21.6)		115 (22.3)	
Adjuvant Therapy (%)					0.712
No	1138 (69.1)	499 (70.8)	0.444	364 (70.5)	
Yes	509 (30.9)	206 (29.2)		152 (29.5)	

a means comparing the training cohort with the internal validation group.

b means comparing the derivation set with the external validation group. Data are expressed as numbers (percentages) of participants, unless otherwise indicated.

BMI, Body Mass Index; ASA physical status, American Society of Anesthesiologists physical status; CEA: Carcinoembryonic Antigen; CA125: Cancer Antigen 125.

Regarding survival rates, within the derivation cohort, 537 patients (22.73%) died within 5 years post-surgery. The 5-year OS rates for the training set and internal validation set were 77.61% (75.54%, 79.73%) and 78.64% (75.57%, 81.84%), respectively. The 5-year OS rate for patients in the external validation cohort was 79.95% (76.48%, 83.58%) (**Supplementary Figure 1**). **Supplementary Table S1** presents the comparison data of patients who died within 5 years post-surgery and those who survived, in both the derivation and external validation cohorts (**Supplementary Table 1**).

### Creating a novel oxidative stress score

Within the training set, the optimal threshold values for oxidative stress markers were identified as follows: albumin (ALB) 39.93 g/dL, total bilirubin (TBIL) 7.77 μmol/L, direct bilirubin (DBIL) 3.01 μmol/L, urea (BUN) 6 mg/dL, uric acid (UA) 296.1 μmol/L, lactate dehydrogenase (LDH) 222 IU/L, and creatinine (Crs) 99.33 μmol/L. Stepwise multivariate Cox regression analysis was utilized to identify the best performing prediction model with the lowest Akaike Information Criterion (AIC) value. Ultimately, six variables with the lowest AIC values were determined: ALB, TBIL, BUN, UA, LDH, and Crs (**Table 2**). Consequently, based on the variable coefficients from the stepwise regression, a prognostic model for lung cancer-related oxidative stress score (OSS) was further developed: OSS=(ALB × (-0.4362)) + (BUN × (-0.2667)) + (TBIL × (-0.3965)) + (UA × 0.3770) + (LDH × (-0.2101)) + (Crs × 0.2679) (**Table 2**). Patients were then stratified into high-risk and low-risk groups according to the optimal cut-off value (OSS=-0.4767978) for the OSS. **Supplementary Table 2** presents the differences in pathological data among different OSS groups within the training set (**Supplementary Table 2**). Kaplan-Meier survival analysis results indicated that patients in the low OSS group had significantly worse survival rates than those in the medium OSS and high OSS groups (*P* < 0.001, **Supplementary Figure 2A**). Survival analysis in the external validation cohort also showed similar results (*P* < 0.001, **Supplementary Figure 2C**), with comparable observations in the internal validation cohort (*P* < 0.294, **Supplementary Figure 2B**).

**Table 2:** Results of stepwise selection of variables based on AIC.

Variable	Coef	Exp(Coef)	Std.Err	z	*P* value
Albumin	-0.4362	0.6465	0.1267	-3.444	0.000574
Urea	-0.2667	0.7659	0.1134	-2.353	0.018632
Total Bilirubin	-0.3965	0.6727	0.1156	-3.43	0.000603
Uric Acid	0.377	1.458	0.1151	3.275	0.001057
Lactate Dehydrogenase	-0.2101	0.8105	0.1401	-1.499	0.133856
Creatinine	0.2679	1.3073	0.1172	2.287	0.022185

Oxidative stress score = (Albumin × (-0.4362)) + (Urea × (-0.2667)) + (Total_Bilirubin × (-0.3965)) + (Uric_Acid × 0.3770) + (Lactate_Dehydrogenase × (-0.2101)) + (Creatinine × 0.2679).

### Variable importance

The Boruta algorithm was employed to process all 36 included clinical and pathological variables in order to reduce data dimensionality and eliminate irrelevant features. Figure **Supplementary Figure 3** displays the output of the Boruta feature selection algorithm. Using this algorithm, 10 features were identified as important, namely: pN, pT, Diameter, CEA, Age, Hemoglobin, CA125, Operation_Time, Neutrophils, and OSS (**Supplementary Figure 3**).

### Model performance: development

**Figure 2:**
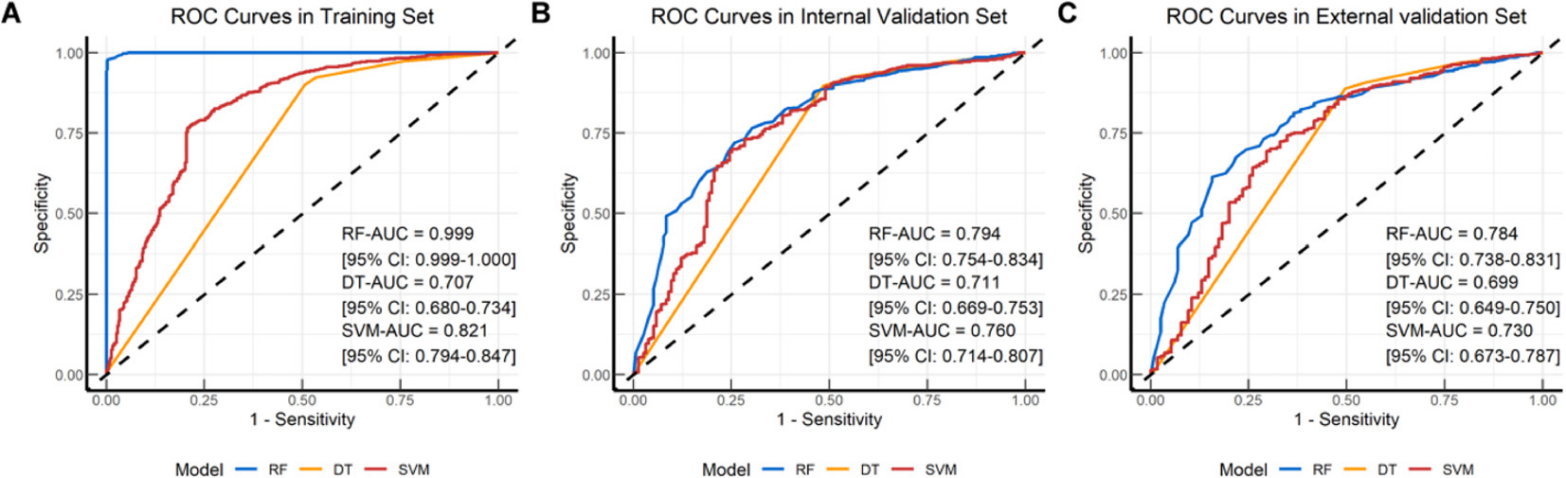
Receiver operating characteristic (ROC) curves plots of the classification models. ROC curve plot in the **(A)** Training set; **(B)** Internal validation set; **(C)** External validation set. RF, Random Forest; DT, Decision Tree; SVM, Support Vector Machine.

We incorporated OSS along with 9 other variables into machine learning, constructing three different models (RF, DT, SVM). **Figure 2** displays the receiver operating characteristic curves and AUC values for these models in predicting 5-year follow-up mortality across the training set, internal validation set, and external validation set (**Figure 2**). The AUC for the RF model was 0.999 (95% CI: 0.999-1.000), 0.794 (95% CI: 0.754-0.834), and 0.784 (95% CI: 0.738-0.831) for the training, internal validation, and external validation sets, respectively. For the DT model, the AUC values were 0.707 (95% CI: 0.680-0.734), 0.711 (95% CI: 0.669-0.753), and 0.699 (95% CI: 0.649-0.750) across the same sets. The SVM model had AUC values of 0.821 (95% CI: 0.794-0.847), 0.760 (95% CI: 0.714-0.807), and 0.730 (95% CI: 0.673-0.787). The RF model demonstrated superior AUC values across all datasets compared to the DT and SVM models, indicating excellent predictive performance and strong generalization ability. The minimal variation in AUC values for the RF model across different validation datasets underscores its efficiency and stability in predicting overall survival in elderly lung cancer patients.

### Model performance: calibration and decision curves

**Figure 3:**
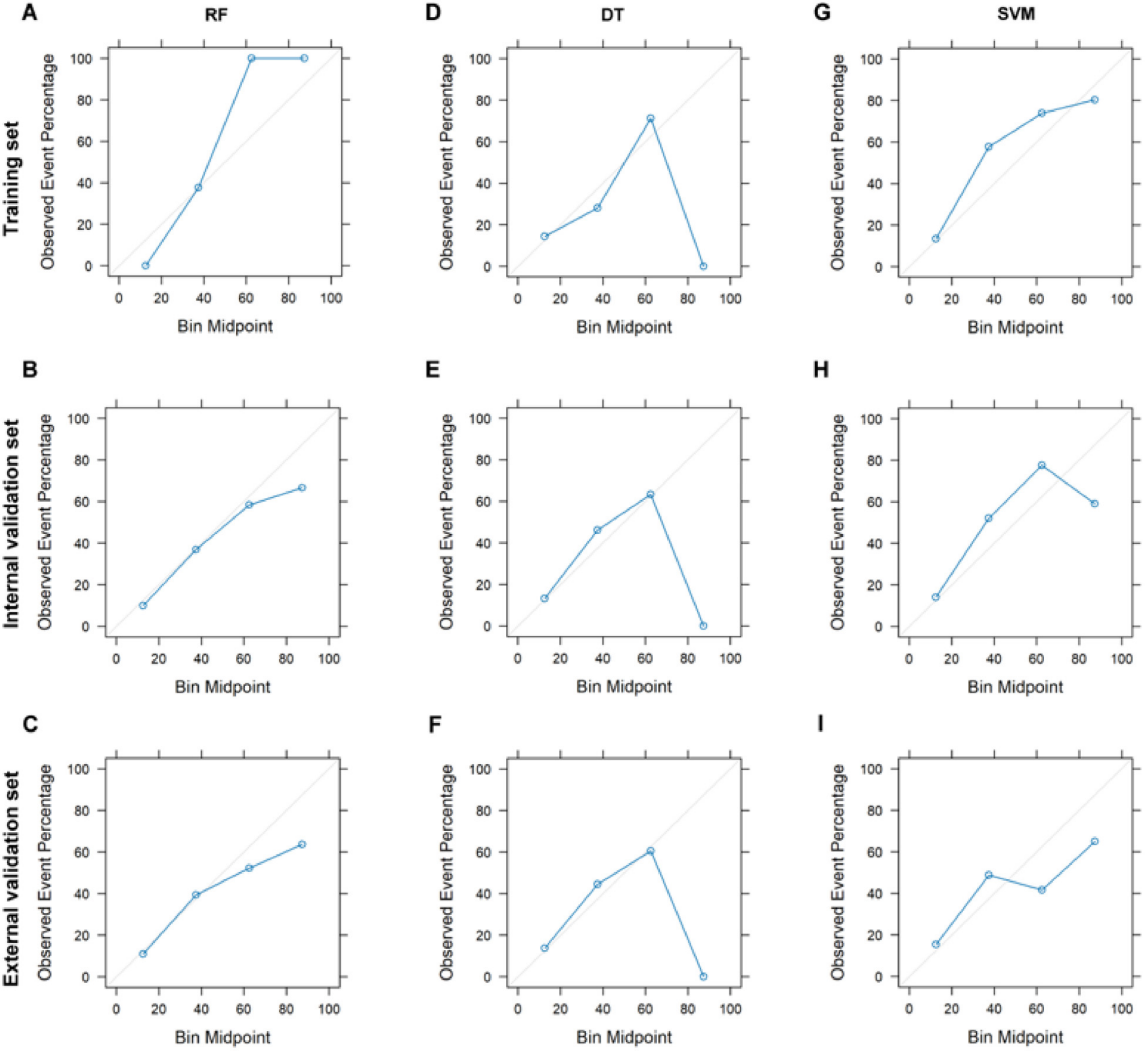
Calibration curves plot for different classification models. Calibration curves for RF model on training **(A)**, internal validation **(B)**, and external validation **(C)** sets. Calibration curves for DT model on training **(D)**, internal validation **(E)**, and external validation **(F)** sets. Calibration curves for SVM model on training **(G)**, internal validation **(H)**, and external validation **(I)** sets. RF, Random Forest; DT, Decision Tree; SVM, Support Vector Machine.

The calibration plots reveal that the RF model consistently aligns predicted probabilities with observed event frequencies, particularly within the medium to low probability range (**Figures 3A–C**), indicating its strong generalizability. Conversely, the DT model’s predictions generally match actual outcomes across most datasets but exhibit slight deviations in the external validation set (**Figures 3D–F**), suggesting potential overfitting and poor generalization to new data. The SVM model’s calibration remains close to the 45° line in both the training and validation sets, with minor deviations in certain probability intervals (**Figures 3G–I**), indicating good calibration and consistent performance across datasets. Additionally, decision curve analysis was used to assess the clinical utility of these models. The RF model demonstrated the highest net benefit across most threshold probabilities in the training set, while the DT and SVM models showed similar net benefits, both lower than that of the RF model (**Supplementary Figure 4A**). In the internal validation set, the RF model continued to show higher net benefit across most thresholds, with DT and SVM performing similarly and both lower than RF (**Supplementary Figure 4B**). Even though performance declined for all models in the external validation set, RF still outperformed DT and SVM across most thresholds (**Supplementary Figure 4C**).

## Discussion

As a distinct population, elderly individuals, due to their unique biopsychosocial characteristics, may be at increased risk when undergoing surgery for lung cancer, facing greater challenges such as more comorbidities, increased frailty, reduced stress tolerance, decreased physical function, and cognitive decline, making their postoperative survival outcomes subject to more complex factors (16, 17). In assessing the long-term care quality of oncological surgery, the 5-year survival rate following curative surgery for malignancy is an important audit indicator. Consequently, establishing a prognostic model for elderly patients with NSCLC can aid in guiding individualized treatment and follow-up strategies for this demographic. By developing machine learning models (DT, RF, SVM), this study has effectively predicted the 5-year survival rate of elderly lung cancer patients following surgery. The RF model demonstrated superior performance, achieving AUC values of 0.794 (95% CI: 0.754-0.834) and 0.784 (95% CI: 0.738-0.831) in the validation cohorts. This model can make individualized predictions about postoperative survival for elderly NSCLC patients based on their clinical and pathological data, thereby enabling targeted follow-up strategies for patients. This includes shortening or extending the intervals between follow-ups, adding or omitting items from the follow-up schedule, which can alleviate the economic burden on patients and society, and also allows for the timely detection of risk factors affecting patient survival and their active treatment.

Previous clinical studies have preliminarily established the predictive value of specific clinical-pathological biomarkers in the recurrence, metastasis, and overall survival of lung cancer post-surgery. These biomarkers include tumor size, differentiation status, inflammatory markers, and TNM staging (18–20). Despite this, these predictive biomarkers fail to reflect the complex prognostic situation of elderly lung cancer patients. As for oxidative stress, it catalyzes glycolysis, stimulates tumor cell migration, and enhances tumor growth (21). Additionally, oxidative stress has been associated with overexpression of ferritin metabolic genes, thereby interfering with prognosis (22). Studies in animal models have shown that oxidative stress factors rise following external stimuli in mice, leading to significant increases in biochemical markers such as TBIL, LDH, creatinine, and BUN, which can promote tumorigenesis and development (23, 24). In elderly patients with malignant tumors, the oxidative stress process is often imbalanced, potentially affecting the migration and invasion capabilities of malignant tumor cells and possibly impacting the prognosis of these patients, though long-term prognostic studies have not yet been reported. Building on this, our study introduces the lung cancer oxidative stress indicator OSS, which includes ALB, TBIL, BUN, UA, LDH, and Crs, all closely linked to oxidative stress. Our results show that patients with low OSS have a poorer prognosis compared to those with high OSS. The OSS was formulated by training on a cohort of patients from our institution, utilizing detailed clinical data and extended follow-up. Consequently, we hypothesize that a predictive model incorporating OSS could more accurately forecast the prognosis of elderly lung cancer patients.

Previous reports have described several models for predicting postoperative survival in lung cancer patients. Larsen A developed a model based on a general inflammatory score, exploring the prognostic value of albumin, C-reactive protein, neutrophil count, lymphocyte count, hemoglobin, and the neutrophil-to-lymphocyte ratio (NLR) for NSCLC through a non-machine learning model (25). However, this study was limited by its inclusion of only hematological indicators, lacking the generalizability and automation offered by machine learning, potentially missing critical prognostic factors. To overcome this limitation, She and colleagues included a more comprehensive set of 127 features, encompassing patient characteristics, tumor staging, and treatment strategies, and established a deep learning model. This survival neural network model demonstrated better results in predicting lung cancer-specific survival compared to tumor, lymph node, and metastasis stages with a C index of 0.739, both in internal modeling and external validation cohorts (26). However, this model did not stratify elderly patients separately. Ganti used data from 38 centers on lung cancer cases to create a predictive model for the overall survival of elderly NSCLC patients, finding that male gender, poor performance status, distant metastasis, and recent weight loss were reasons for poorer prognosis in this group, with an area under the ROC curve for 1-year and 2-year OS prediction of 0.6 and 0.65, respectively (27). However, this model suffered from an inability to reflect the physiological characteristics of the elderly adequately, and the predictive efficiency of the model was low. Similarly, for prognosis prediction in elderly NSCLC patients, Wang and colleagues used frailty indices, indicators reflecting the physiological state and general pathological response of the elderly, to evaluate prognosis in elderly lung cancer patients, demonstrating that frail patients had a higher overall risk of mortality and higher prognostic value for survival (AUC range = A) (28). However, this study was limited to single-center data and a median follow-up time of less than two years, potentially limiting the broader application of the model. In this study, various machine learning models were developed and validated to enhance the predictive accuracy for 5-year overall survival (OS) in elderly NSCLC patients. The RF model exhibited superior performance compared to the other models, with excellent calibration and predictive capabilities. Our model leverages commonly available perioperative clinical data, focusing on seven critical variables influencing 5-year postoperative survival: pT, pN, tumor location, OSS, tumor size, degree of differentiation, and perineural invasion. This approach underscores the significance of preoperative oxidative stress, overall systemic health, surgical performance, postoperative recovery, and tumor staging in predicting long-term survival rates in elderly lung cancer patients. Decision curve analysis was utilized to compare the clinical utility of the different models across various thresholds, further aiding in model selection and application. The RF model consistently demonstrated higher net benefits across training, testing, and validation datasets, suggesting robust generalizability and effectiveness in clinical practice.

In numerous studies, RF models have demonstrated superior performance compared to DT and SVM models, primarily attributed to their unique structure and algorithmic characteristics (29). The ensemble learning approach inherent to RF models confers robust feature processing capabilities, enabling excellent performance in handling high-dimensional data and feature selection (30). This ensemble method also enhances the model’s stability and generalization ability. These advantages not only contribute to the RF model’s exceptional accuracy but also allow it to maintain stable performance across various complex application scenarios (31). Our innovative RF model achieved a higher AUC value than previous models, likely due to its incorporation of a broader range of clinical evaluation indicators for elderly patients, such as oxidative stress markers, age-adjusted comorbidity indices, and comprehensive complication indices. Variables pN and pT emerged as the most critical for model prediction, with their significance surpassing that of other variables. Hemoglobin, tumor markers, OSS, and tumor size also demonstrated considerable importance. These findings highlight the key factors that influence the model’s performance, facilitating its further optimization and interpretation. Consequently, when developing a model for predicting long-term survival after lung cancer surgery, it is crucial to consider these prognostic factors comprehensively.

We must acknowledge certain limitations of our study. The retrospective design precluded the collection of more specialized oxidative stress indicators, such as superoxide dismutase, malondialdehyde, and redox potential, while our retrospective design limited the biomarkers we could include, we acknowledge the value of additional markers, we expect that more potential biomarkers that could be included in future studies to strengthen the OSS and enhance the model’s predictive power. Additionally, our database did not include other significant factors influencing lung cancer, such as high-risk gene mutations, immunotherapy usage, and socioeconomic status, which could impact model performance. We hope that future multicenter, large-sample, and multi-ethnic studies can further enhance the model’s applicability.

## Conclusions

The clinical prediction model based on OSS and developed using machine learning techniques demonstrates effective prognostic capabilities for elderly lung cancer patients following curative surgery.

## Data Availability

The original contributions presented in the study are included in the article/**Supplementary Material**. Further inquiries can be directed to the corresponding author.
