# Supplementary material for: Machine learning-based prediction of 5-year survival in elderly NSCLC patients using oxidative stress markers
Source: Front Oncol. 2024 Oct 24;14:1482374. doi: 10.3389/fonc.2024.1482374 (PMC11540553; doi:10.3389/fonc.2024.1482374)
Supplement: Supplementary file 1 [file DataSheet1.docx]

eFigure 1. Survival curves were generated by Kaplan-Meier survival analysis.


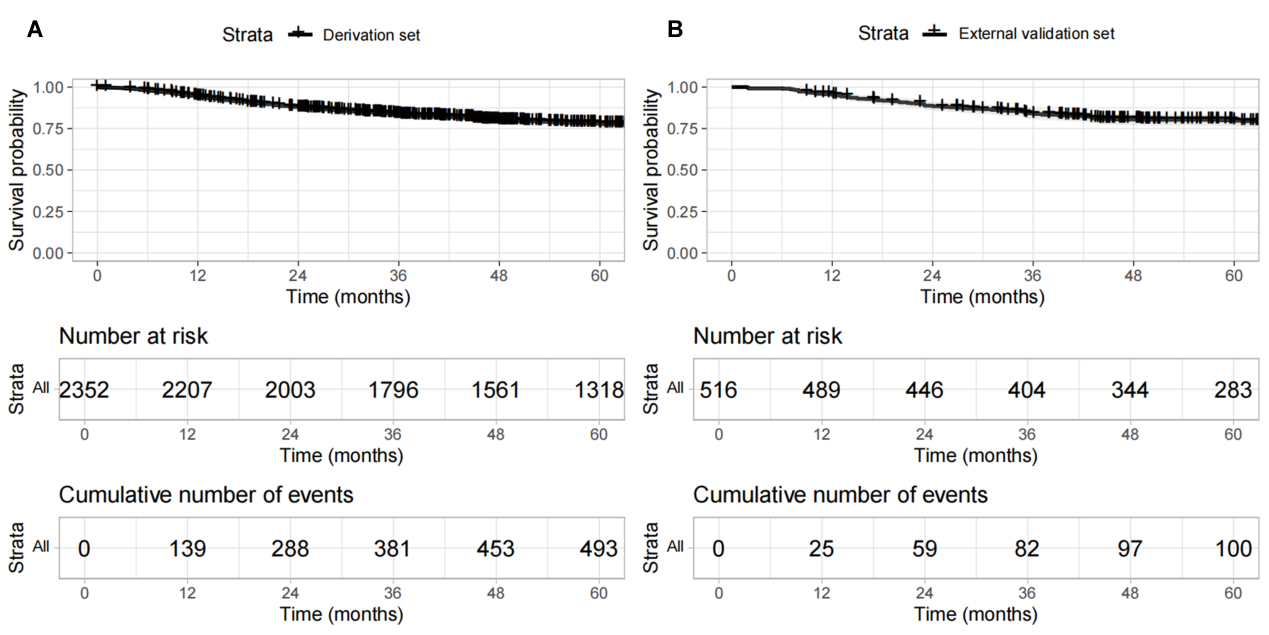


A.Derivation set. B. External validation set

eFigure 2. KM survival curve of oxidative stress score risk.


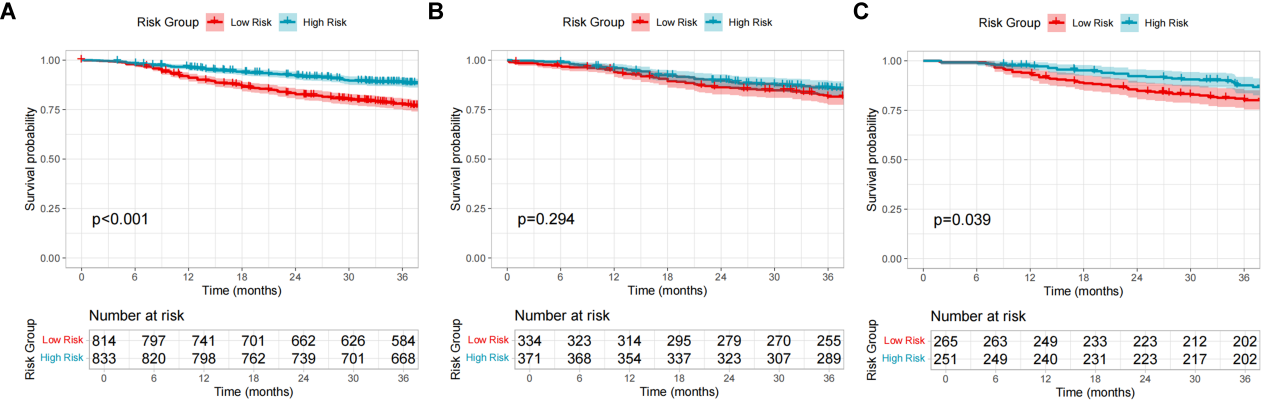


A.Training set. B.Internal validation set. C.External validation set.

eFigure 3.Boruta algorithm was used for feature selection and to assess the importance of variables.


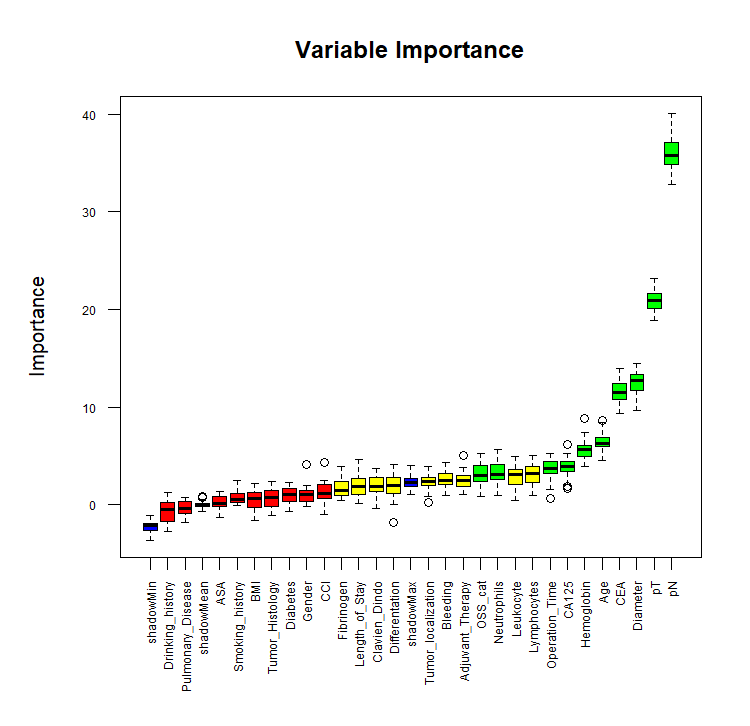


Abbreviations: BMI, Body Mass Index; ASA physical status, American Society of Anesthesiologists physical status; CEA: Carcinoembryonic Antigen; CA125: Cancer Antigen 125; OSS, Oxidative Stress Score; CCI: Charlson Comorbidity Index.

eFigure 4. Decision curve plot for different competitive models.


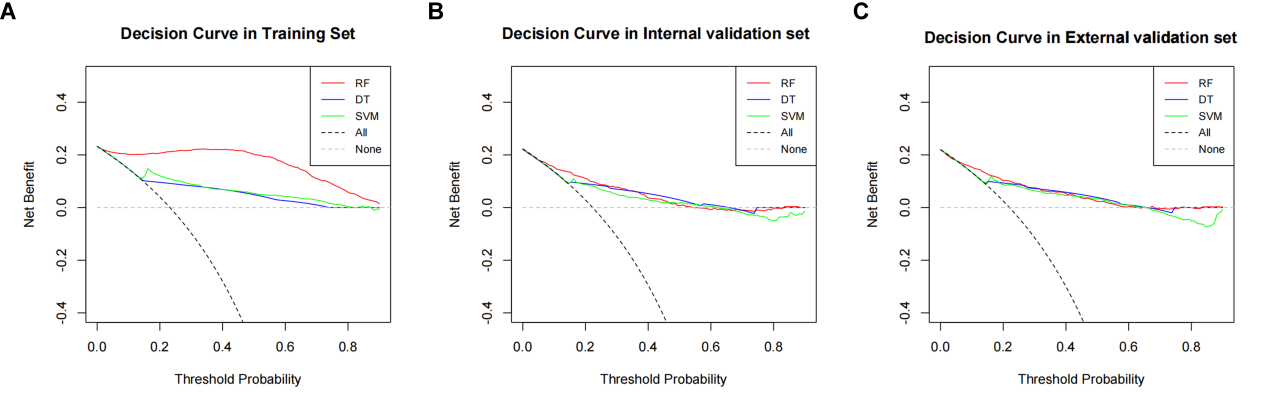


Abbreviations: RF, Random Forest; DT, Decision Tree; SVM, Support Vector Machine.

**eTable1.** Association between different potential risk factors of 5-year mortality in the derivation cohort.

|  | Derivation set | | | External validation set | | |  |
| --- | --- | --- | --- | --- | --- | --- | --- |
|  | Alive ＞ 5 years | Deceased ≤ 5 years | *P* value | Alive ＞ 5 years | Deceased ≤ 5 years | *P* value |  |
| n | 1815 | 537 |  | 401 | 115 |  |  |
| Age (mean (SD)) | 70.97 (4.96) | 72.57 (5.64) | <0.001 | 71.29 (5.15) | 72.66 (5.10) | 0.012 |  |
| Gender (%) |  |  | <0.001 |  |  | 0.816 |  |
| female | 795 (43.8) | 189 (35.2) |  | 164 (40.9) | 45 (39.1) |  |  |
| male | 1020 (56.2) | 348 (64.8) |  | 237 (59.1) | 70 (60.9) |  |  |
| BMI (mean (SD)) | 22.51 (3.44) | 22.23 (3.17) | 0.089 | 22.71 (3.23) | 21.33 (3.07) | <0.001 |  |
| ASA (%) |  |  | 0.047 |  |  | 0.292 |  |
| 1 | 76 (4.2) | 11 (2.0) |  | 13 (3.2) | 5 (4.3) |  |  |
| 2 | 1300 (71.6) | 377 (70.2) |  | 291 (72.6) | 73 (63.5) |  |  |
| 3 | 436 (24.0) | 147 (27.4) |  | 93 (23.2) | 36 (31.3) |  |  |
| 4 | 3 (0.2) | 2 (0.4) |  | 4 (1.0) | 1 (0.9) |  |  |
| Smoking history (%) |  |  | 0.905 |  |  | 0.049 |  |
| No | 1318 (72.6) | 392 (73.0) |  | 285 (71.1) | 70 (60.9) |  |  |
| Yes | 497 (27.4) | 145 (27.0) |  | 116 (28.9) | 45 (39.1) |  |  |
| Pulmonary Disease (%) |  |  | 0.641 |  |  | 1 |  |
| No | 1762 (97.1) | 524 (97.6) |  | 384 (95.8) | 110 (95.7) |  |  |
| Yes | 53 (2.9) | 13 (2.4) |  | 17 (4.2) | 5 (4.3) |  |  |
| CEA (mean (SD)) | 8.53 (53.51) | 27.89 (100.64) | <0.001 | 12.77 (13.50) | 15.96 (18.93) | 0.001 |  |
| CA125 (mean (SD)) | 12.82 (23.89) | 23.59 (121.63) | <0.001 | 3.82 (2.31) | 5.16 (2.36) | 0.043 |  |
| Diameter (mean (SD)) | 3.86 (2.24) | 5.56 (2.79) | <0.001 | 189.74 (53.71) | 192.61 (58.79) | <0.001 |  |
| pT (%) |  |  | <0.001 |  |  | <0.001 |  |
| 1 | 873 (48.1) | 59 (11.0) |  | 108 (26.9) | 37 (32.2) |  |  |
| 2 | 471 (26.0) | 160 (29.8) |  | 81 (20.2) | 59 (51.3) |  |  |
| 3 | 434 (23.9) | 277 (51.6) |  | 3 (0.7) | 5 (4.3) |  |  |
| 4 | 37 (2.0) | 41 (7.6) |  | 305 (76.1) | 34 (29.6) |  |  |
| pN (%) |  |  | <0.001 |  |  | <0.001 |  |
| 0 | 1340 (73.8) | 161 (30.0) |  | 47 (11.7) | 22 (19.1) |  |  |
| 1 | 282 (15.5) | 107 (19.9) |  | 36 (9.0) | 34 (29.6) |  |  |
| 2 | 145 (8.0) | 147 (27.4) |  | 13 (3.2) | 25 (21.7) |  |  |
| pTNM (%) |  |  | <0.001 |  |  | <0.001 |  |
| Ⅰ | 824 (45.4) | 46 (8.6) |  | 200 (49.9) | 8 (7.0) |  | 1 |
| Ⅱ | 666 (36.7) | 147 (27.4) |  | 137 (34.2) | 36 (31.3) |  |  |
| Ⅲ | 325 (17.9) | 344 (64.1) |  | 64 (16.0) | 71 (61.7) |  |  |
| Tumor Histology (%) |  |  | 0.066 |  |  | 0.325 |  |
| Adenocarcinoma | 1332 (73.4) | 380 (70.8) |  | 95 (23.7) | 20 (17.4) |  |  |
| Adenosquamous carcinoma | 83 (4.6) | 38 (7.1) |  | 285 (71.1) | 79 (68.7) |  |  |
| Squamous carcinoma | 400 (22.0) | 119 (22.2) |  | 116 (28.9) | 36 (31.3) |  |  |
| Adjuvant Therapy (%) |  |  | 0.65 |  |  | 0.706 |  |
| No | 1268 (69.9) | 369 (68.7) |  | 12.88 (6.35) | 14.21 (6.67) |  |  |
| Yes | 547 (30.1) | 168 (31.3) |  | 197 (49.1) | 68 (59.1) |  |  |

Abbreviations: BMI, Body Mass Index; ASA physical status, American Society of Anesthesiologists physical status; CEA: Carcinoembryonic Antigen; CA125: Cancer Antigen 125.

eTable 2. The relationship between oxidative stress score and clinical features in the training set.

|  | High risk | Low risk | *P* value |
| --- | --- | --- | --- |
| n | 814 | 833 |  |
| Age (mean (SD)) | 71.93 (5.37) | 70.79 (4.85) | <0.001 |
| Gender (%) |  |  | 0.177 |
| female | 327 (40.2) | 363 (43.6) |  |
| male | 487 (59.8) | 470 (56.4) |  |
| BMI (mean (SD)) | 22.61 (3.33) | 22.15 (3.20) | 0.004 |
| ASA (%) |  |  | 0.116 |
| 1 | 24 (2.9) | 41 (4.9) |  |
| 2 | 600 (73.7) | 579 (69.5) |  |
| 3 | 189 (23.2) | 212 (25.5) |  |
| 4 | 1 (0.1) | 1 (0.1) |  |
| Smoking history (%) |  |  | 0.289 |
| No | 581 (71.4) | 615 (73.8) |  |
| Yes | 233 (28.6) | 218 (26.2) |  |
| Pulmonary Disease (%) |  |  | 0.703 |
| No | 794 (97.5) | 809 (97.1) |  |
| Yes | 20 (2.5) | 24 (2.9) |  |
| CEA (mean (SD)) | 15.34 (80.64) | 11.89 (62.55) | 0.332 |
| CA125 (mean (SD)) | 14.53 (23.99) | 16.79 (97.06) | 0.518 |
| Diameter (mean (SD)) | 4.72 (2.63) | 3.79 (2.31) | <0.001 |
| pT (%) |  |  | <0.001 |
| 1 | 274 (33.7) | 377 (45.3) |  |
| 2 | 238 (29.2) | 204 (24.5) |  |
| 3 | 267 (32.8) | 225 (27.0) |  |
| 4 | 35 (4.3) | 27 (3.2) |  |
| pN (%) |  |  | <0.001 |
| 0 | 472 (58.0) | 569 (68.3) |  |
| 1 | 138 (17.0) | 144 (17.3) |  |
| 2 | 121 (14.9) | 80 (9.6) |  |
| 3 | 83 (10.2) | 40 (4.8) |  |
| pTNM (%) |  |  | <0.001 |
| Ⅰ | 251 (30.8) | 354 (42.5) |  |
| Ⅱ | 283 (34.8) | 282 (33.9) |  |
| Ⅲ | 280 (34.4) | 197 (23.6) |  |
| Tumor Histology (%) |  |  | 0.021 |
| Adenocarcinoma | 588 (72.2) | 609 (73.1) |  |
| Adenosquamous carcinoma | 53 (6.5) | 30 (3.6) |  |
| Squamous carcinoma | 173 (21.3) | 194 (23.3) |  |
| Adjuvant Therapy (%) |  |  | 0.826 |
| No | 565 (69.4) | 573 (68.8) |  |
| Yes | 249 (30.6) | 260 (31.2) |  |

Abbreviations: BMI, Body Mass Index; ASA physical status, American Society of Anesthesiologists physical status; CEA: Carcinoembryonic Antigen; CA125: Cancer Antigen 125.
